# Supplementary material for: Reproducibility of Electromagnetic Field Simulations of Local Radiofrequency Transmit Elements Tailored for 7 T MRI
Source: Sensors (Basel). 2025 Mar 17;25(6):1867. doi: 10.3390/s25061867 (PMC11945818; doi:10.3390/s25061867)
Supplement: Supplementary file 1 [file sensors-25-01867-s001.zip › sensors-3475451-supplementary.pdf]

Supporting Information

Meshing Overview

The meshing techniques of the different simulation solvers resulted in varying meshes. This is based on the different meshing engines and mesh types. Supporting Figure 1 to Supporting Figure 4 show the mesh settings for the four Tx elements of each simulation tool (CST Microwave Studio, HFSS, Sim4Life) as well as the used simulation settings.

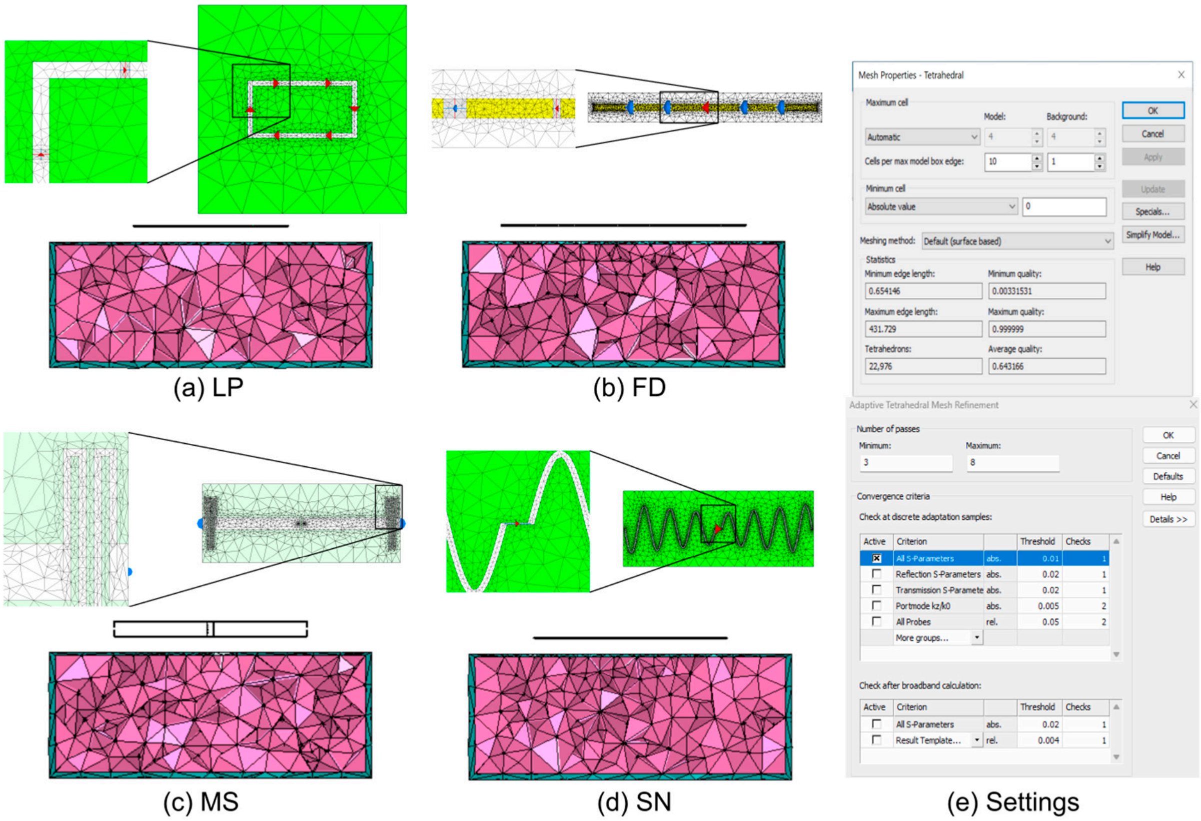

Supporting Figure S1: Mesh view of the a) Loop (LP), b) Fractionated Dipole (FD), c) Microstrip with meanders (MS), and d) Snake antenna (SN) on a rectangular phantom using the finite element method (FEM) with tetrahedral mesh cells in CST Microwave Studio 2020. e) Global mesh settings and the adaptive mesh refinement settings are shown.

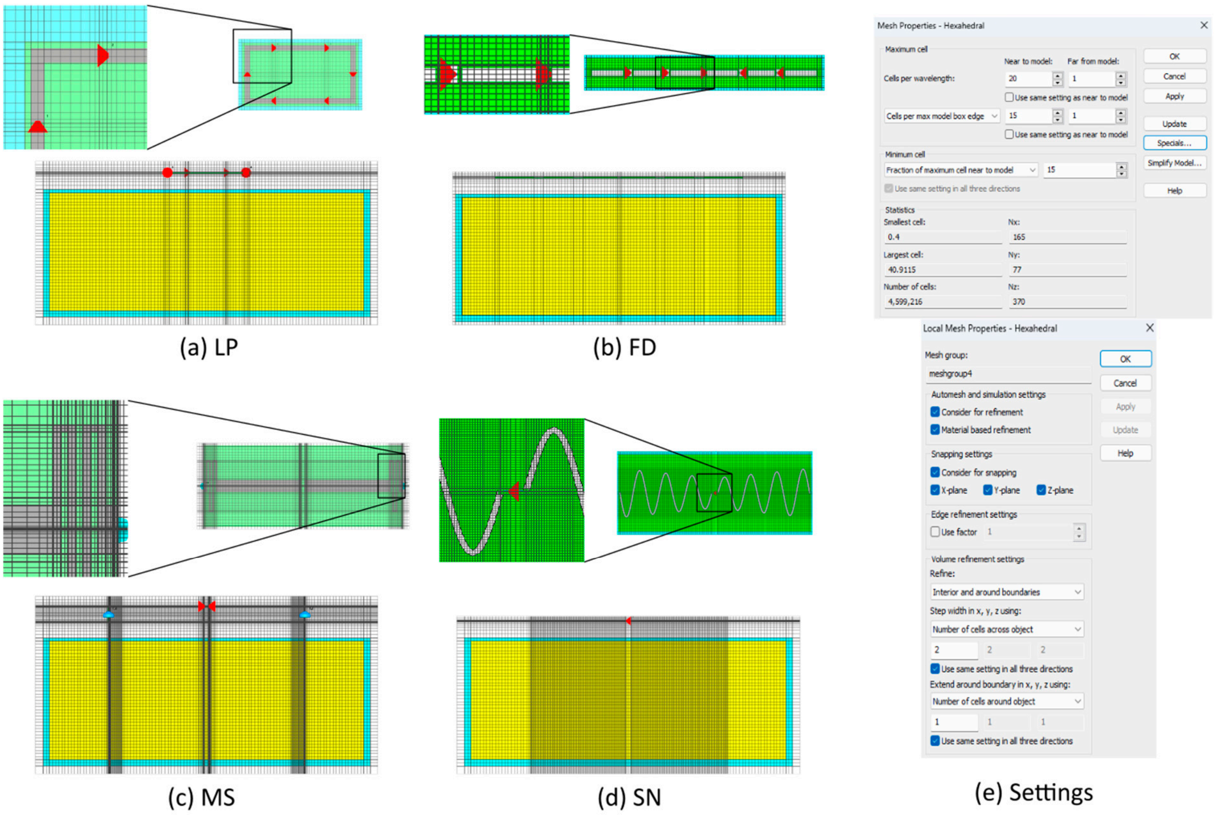

Supporting Figure S2: Mesh view of the a) Loop (LP), b) Fractionated Dipole (FD), c) Microstrip with meanders (MS), and d) Snake antenna (SN) on a rectangular phantom using the finite integration technique (FIT) with hexahedral mesh cells in CST Microwave Studio 2022. e) Global mesh settings and the adaptive mesh refinement settings are shown.

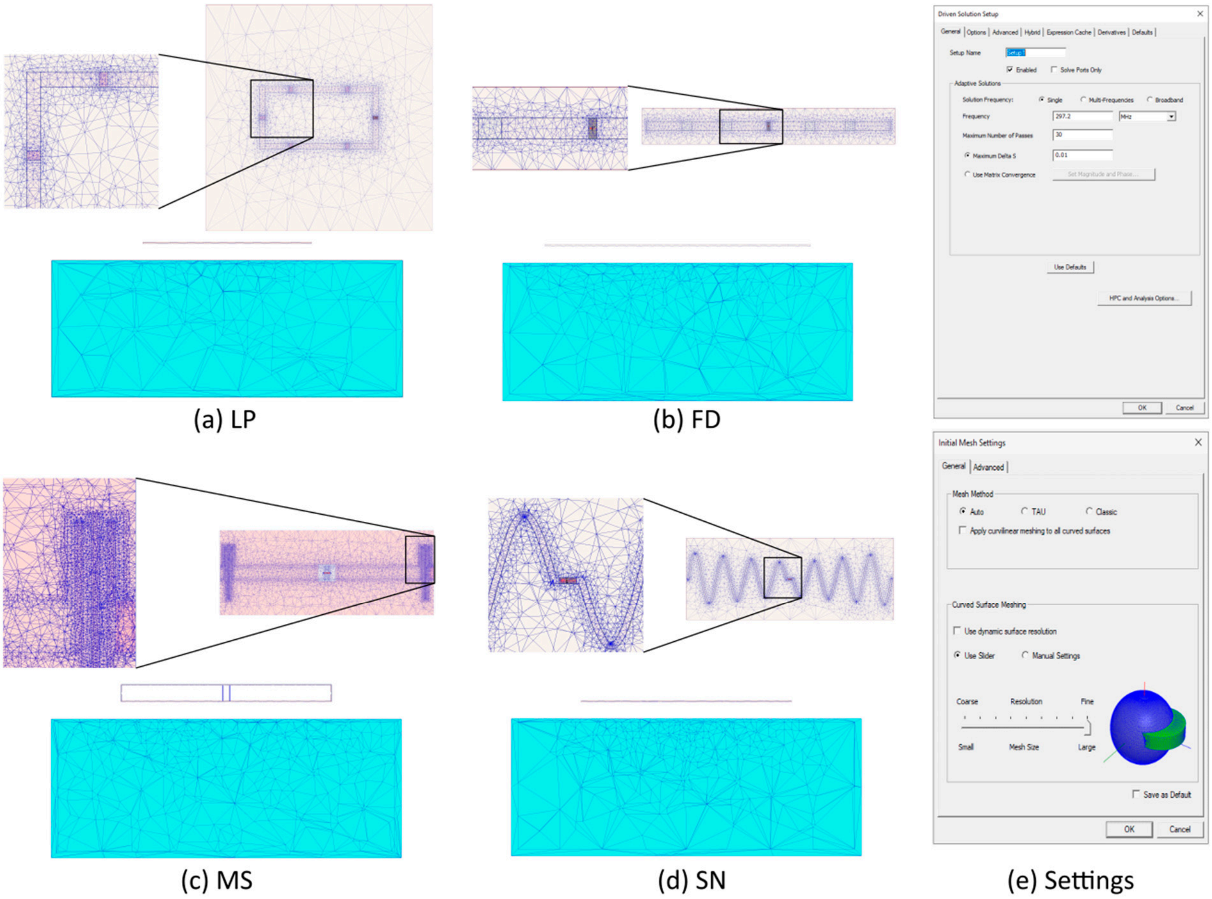

Supporting Figure S3: Mesh view of the a) Loop (LP), b) Fractionated Dipole (FD), c) Microstrip with meanders (MS), and d) Snake antenna (SN) on a rectangular phantom using the finite element method (FEM) with tetrahedral meshes in HFSS 2021R1. e) Global mesh settings and the adaptive mesh refinement settings are shown.

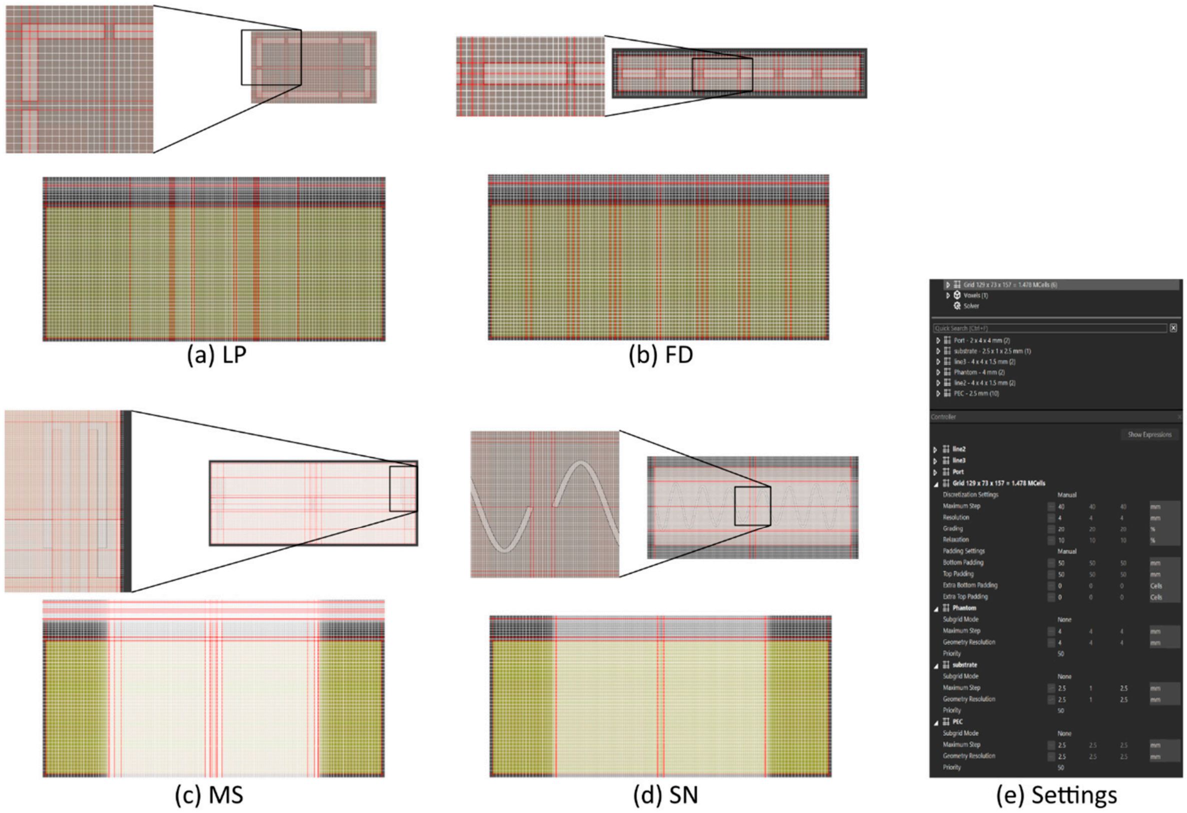

Supporting Figure S4: Mesh view of the a) Loop (LP), b) Fractionated Dipole (FD), c) Microstrip with meanders (MS), and d) Snake antenna (SN) on a rectangular phantom using the finite-difference time-domain (FDTD) with hexahedral mesh cells in Sim4Life V8.0. E) Mesh (grid) settings are shown.

Guideline for the Co-Simulation (Post-Processing)

The co-simulations for tuning and matching each Tx element were performed in post-processing. Here the different simulation tools vary in how this is implemented. Where HFSS and CST have a dedicated schematic tool where the tuning and matching networks in addition to the 3D model results can be included and used as a circuit simulation, Sim4Life only provides specific tuning and matching options without a dedicated circuit simulator. Supporting Figure S5 shows, how the tuning and matching was performed in the three simulation environments. In CST the capacitors can be modeled with parasitic components whereas in HFSS, the parasitic components must be added as dedicated elements. In Sim4Life no losses were considered because the resistors could only be modeled for the serial elements not for parallel elements.

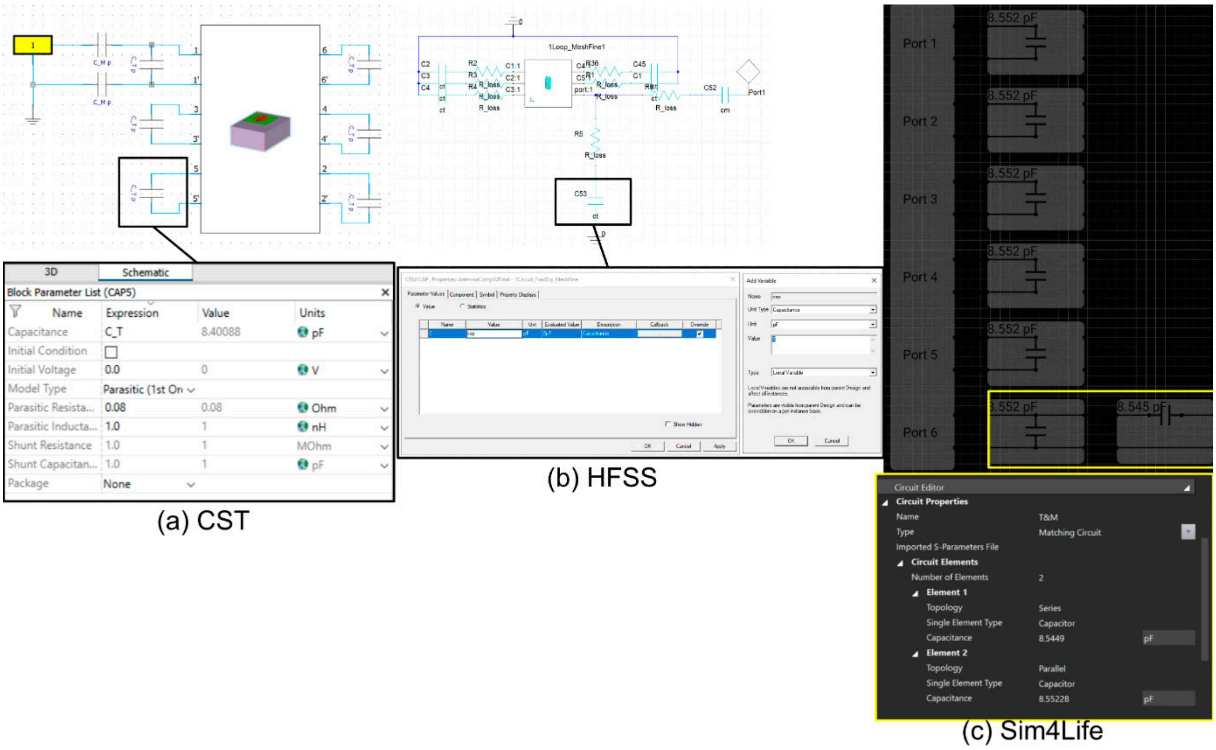

Supporting Figure S5: Modeling of the tuning and matching network of the loop in a) CST Studio 2020, b) HFSS 2021R1, and c) Sim4Life V8.0. The loop was simulated with 6 ports and afterward in the post-processing, each port was assigned to a tuning capacitor or to a tuning and matching network with an excitation port.

Decoupling

The decoupling results of two adjacent elements represented by $|S_{21}|$ is depicted in Supporting Figure S6. It shows, that at the Larmor frequency of 297.2 MHz all simulations tools show similar behavior. Only towards the boundaries of the simulated frequencies the deviation increased.

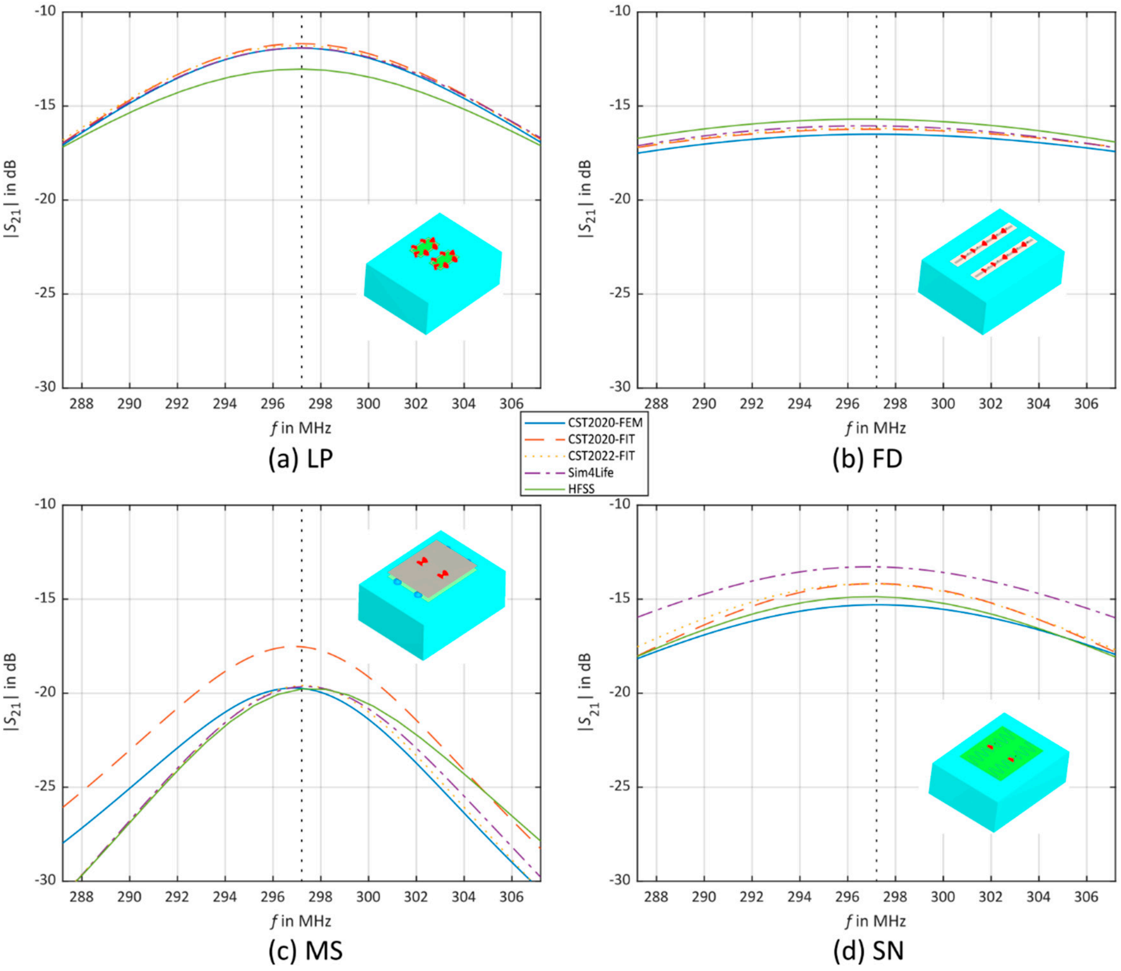

Supporting Figure S6: Decoupling ($|S_{21}|$) between two neighboring a) loop (LP), b) fractionated dipole (FD), c) microstrip with meanders, and d) snake antenna with 100 mm center-to-center distance. *S*-parameters are shown from 287.2 MHz to 307.2 MHz with a target frequency of 297.2 MHz (dashed line*).*

Alternative Tuning & Matching Networks

The balun of the snake antenna and the coaxial transmission line of the microstrip antenna could not be implemented in Sim4Lifes tuning and matching engine. Therefore, work arounds were implemented. Both were tuned and matched using a simple L-network. The microstrip antenna was excited by two ports whereby the second port had a 180° phase shift to mimic the transmission line. The alternative tuning and matching of these two elements is shown in Supporting Figure S7.

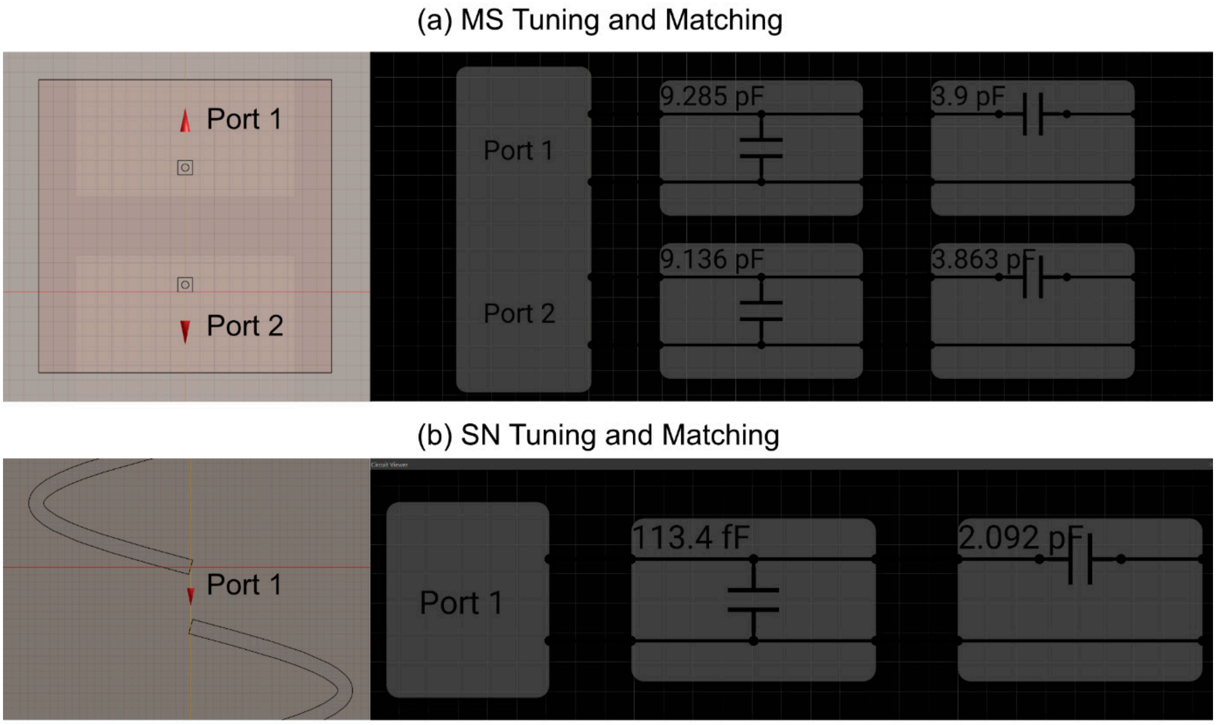

Supporting Figure S7: Tuning and matching network of the a) microstrip with meanders, and b) snake antenna using a parallel and a serial capacitor in Sim4Life V8.0. In the circuit editor, the lumped elements can only be aligned in a row which does not allow any cross-connection between lumped elements.
